# Quantitative investigation of the poly-adenine DNA dissociation from the surface of gold nanoparticles

**DOI:** 10.1038/srep10158

**Published:** 2015-05-14

**Authors:** Weiwen Lu, Lihua Wang, Jiang Li, Yun Zhao, Ziang Zhou, Jiye Shi, Xiaolei Zuo, Dun Pan

**Affiliations:** 1 Key Laboratory of Bio-Resources and Eco-Environment, Ministry of Education, College of Life Sciences, Sichuan University, Chengdu, Sichuan 610064, China; 2 Laboratory of Physical Biology, Shanghai Institute of Applied Physics, Chinese Academy of Sciences, Shanghai 201800, China; 3 Johns Hopkins University, 3400 North Charles Street, Baltimore, Maryland 21218, US; 4 UCB Pharma, Slough, UK

## Abstract

In recent years, poly adenine (polyA) DNA functionalized gold nanoparticles (AuNPs) free of modifications was fabricated with high density of DNA attachment and high hybridization ability similar to those of its thiolated counterpart. This nanoconjugate utilized poly adenine as an anchoring block for binding with the AuNPs surface thereby facilitated the appended recognition block a better upright conformation for hybridization, demonstrating its great potential to be a tunable plasmonic biosensor. It’s one of the key points for any of the practical applications to maintaining stable conjugation between DNA oligonucleotides and gold nanoparticles under various experimental treatments. Thus, in this research, we designed a simple but sensitive fluorescence turn-on strategy to systematically investigate and quantified the dissociation of polyA DNA on gold nanoparticles in diverse experimental conditions. DNA desorbed spontaneously as a function of elevated temperature, ion strength, buffer pH, organic solvents and keeping time. What’s more, evaluating this conjugate stability as affected by the length of its polyA anchor was another crucial aspect in our study. With the improved understanding from these results, we were able to control some of our experimental conditions to maintain a good stability of this kind of polyA DNA−AuNPs nanoconjugates.

DNA-functionalized gold nanoparticles (AuNPs) which integrated the molecular recognition and self-assembly ability of DNA^1^,^2^,^3^,^4^,^5^ with the high extinction coefficient and excellent optical properties of AuNPs have played a vital role in the flourishing field of nanotechnology since 1996^6^,^7^. Meanwhile, numerous of fundamental insights have been obtained by using such versatile nanoconjugates, which demonstrated their appealing perspective and great potential for various applications^8^,^9^,^10^,^11^,^12^,^13^,^14^,^15^,^16^,^17^. They have been employed extensively for gene regulation^18^,^19^,^20^, drug delivery^21^,^22^,^23^,^24^, novel nanostructure fabrication^3^,^6^, optimization of the specificity of polymerase chain reactions (PCR)^25^, biosensoring for metal ions, small molecules, biomolecules and even living cells^26^,^27^,^28^,^29^,^30^,^31^,^32^,^33^,^34^,^35^. AuNPs are stable, catalytically active^36^, and can be readily synthesized by the simple reduction chemistry^31^. Moreover, AuNPs possess high extinction coefficients and a distance dependent optical property^27^,^37^. Traditional DNA−AuNPs nanoconjugates exploit strong Au−S bond to assemble thiolated oligonucleotides at the surface of AuNPs^6^,^27^. Owing to the protection of the dense DNA layer, AuNPs could remain more favorable colloidal stability. Nevertheless, despite the promise of such thiolated DNA AuNPs conjugates in many applications, it’s still difficult to precisely control the orientation and density of such thiolated oligonucleotides and tune the hybridization ability. In addition, the dissociation of DNA which derived from the Au−S bond cleavage would happen easily when the conjugates were exposed to other thiols, high temperatures and acidic buffer solutions^38^,^39^. Since stable linkage between DNA and AuNPs means a lot to most applications, researchers utilized different organosulfur anchors such as alkanethiol, acyclic disulfide and cyclic disulfide for conjugation^40^,^41^,^42^. Studies focused on the salt-dependent colloidal stability of DNA−AuNPs^16^,^43^,^44^ and studies about dissociation of thiol-modified DNA−AuNPs in aqueous and organic solvents^45^,^46^ have emerged during these years as well.

Since 2004, researchers have found that single-stranded, short, and unfolded polyA DNA could protect AuNPs against salt-induced aggregation^24^,^29^. Extensive essential investigations were focused on this field. It has been revealed that the polyA DNA loading capacity (with an absorption energy ~120 kJ/mol) is much lower than its thiolated counterpart (~160 kJ/mol). However, polyA DNA seemed to wrap around the AuNPs and failed to obtain an upright conformation^9^,^14^. This phenomenon may explain why few reports exploit this kind of conjugates for biosensing. Lately, two independent investigations have resolved this problem perfectly. Our team reported a new salt-aging strategy that using poly adenine (polyA) as an anchoring block to modified diblock polyA DNA onto the AuNPs^47^. Subsequently, Liu and his co-workers used a low pH method to fabricate polyA poly adenine DNA onto AuNPs with a high dense shell^48^. The two approaches were created conjugates share similar good colloidal stability and facilitated the recognition block an upright conformation that favored DNA hybridization. In our work, we found the surface density decreased along with the length increase of the polyA block. All adenines in the polyA block, independent of the length, are completely adsorbed on AuNPs to enable full surface coverage. We have demonstrated that polyA served as an effective anchoring block and the appended recognition block adopts an upright conformation that favored DNA hybridization. Afterwards, fundamental understanding of the interaction between polyA DNA and AuNPs was demonstrated such as how to control the polarity of polyA DNA adsorption and the optimal number of adenine in the polyA anchor^49^.

In consideration of the costly synthesis of thiol-labeled DNA, it’s necessary to have more fundamental researches about this alternative polyA DNA hybrid and evaluate its possibility for more applications instead of its thiol-labeled counterpart. Numerous of enzymatic molecular biology manipulations such as PCR demand a high stability of the bound DNA. Thus, one primary target is keeping DNA from dissociation^39^. The high salt-dependent colloidal stability and remarkable high temperature tolerance of such DNA−AuNP nanoconjugates were confirmed during previous studies^47^,^48^,^49^. However, few works were directly studied the stability of polyA modified DNA on gold nanoparticles. In this paper, a systematic insight has been interpreted in the kinetics of DNA dissociation as a function of temperature, buffer pH, organic solvent and ionic strength. Three different length of polyA block (polyA_10_, polyA_20_, polyA_30_) with same recognition block were occupied as anchor fragments in our experiment so that conjugate stability with the change of polyA length can be compared apparently. As a consequence, polyA_20_ and polyA_30_ DNA−AuNPs conjugates showed preferable stability in most of the experiment conditions.

## Results and Discussion

To systematically study the dissociation of polyA DNA on golden nanoparticles in diverse experimental conditions and quantify the fraction of released DNA, we designed a set of diblock single-stranded DNA with a different length of polyA block (polyA_10_, polyA_20_, polyA_30_) and a same recognition block to modify the AuNPs. As shown in Table S1, particularly, the 5’ termini of those DNA molecules were labeled with the fluorescent dye carboxyfluorescein (FAM). As a consideration of other factors such as DNA degradation may affect the quantification of released amount of DNA, we choose the 5’ termini of the DNA for FAM labelling. Thus all of the fluorescence should stem from DNA desorption. 13 nm diameter gold nanoparticles were selected for our research since these particles were readily synthesized and large enough to quench the fluorescence of bound fluorescent DNA completely.

**Figure 1:**
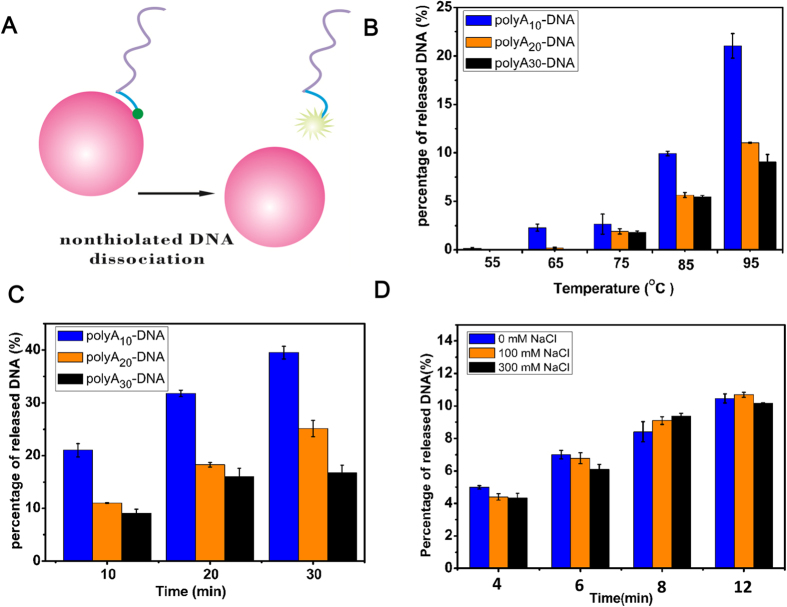
Effect of temperature (B, C) and NaCl concentration (D) on the release of the polyA-DNAs into solution. Each sample also contained 5 mM HEPES, pH 7.6. (A) Schematic illustrating the fluorescence-based measurement of the released FAM-labeled DNA from the DNA−AuNPs. (B) Fraction of released DNA from polyA_10_, polyA_20_, polyA_30_ DNA-AuNPs over a period of 10 min at different temperatures (55, 65, 75, 85 and 95 °C) (C) Fraction of released DNA from polyA_10_, polyA_20_, polyA_30_ DNA-AuNPs over a period of 10, 20 and 30 min at 95 °C (D) Fractions of released DNA from polyA_20_ DNA-AuNPs over a periods of 4, 6, 8 and 12 min at 95 °C in different concentration of NaCl.

We then evaluated the stabilities of these polyA DNA−AuNPs probes by designing a simple but sensitive fluorescence turn-on assay (Fig. 1A) that have been used in the thiolated DNA−AuNPs^38^,^45^,^46^. To put it simply, the fluorescence of FAM-labeled DNA was quenched as a consequence of the close proximity between FAM and AuNPs when the DNA molecule was conjugated to AuNPs. Once the DNA molecule was released from AuNPs, the fluorescence intensity could be monitored easily.

In a typical experiment to functionalize AuNPs, an excess amount of DNA (1.5 μM) was incubated with ~10 nM AuNPs. The hybrids were purified by centrifugation to remove free DNA in the supernatant, and the purified hybrids were resuspended in 5 mM HEPES buffer and stored under various environmental and buffer conditions. At designated time points the AuNPs were centrifuged and the supernatant fluorescence intensity was measured to quantify the fraction of released DNA.

### Thermal desorption of DNA from the DNA**−**AuNPs conjugates

The DNA−AuNPs conjugates were always used in biotechnological experiments that require high temperatures, such as polymerase chain reaction (PCR).As a consequence, on the basis of the fluorescence measuring scheme described above, we studied the effect of variable length of polyA on the thermal stability of DNA−AuNPs conjugates by comparing the fraction of desorbed DNA from those DNA−AuNPs conjugates at elevated temperatures over time. AuNPs modified with those three different DNAs (polyA_10_, polyA_20_ and polyA_30_) were dispersed in 5 mM of HEPES buffer solution at pH 7.4. Heating these DNA-AuNPs hybrids at 55 °C, 65 °C, 75 °C, 85 °C and 95 °C for 10 minutes (Fig. 1B) resulted in fluorescent increases. DNA−AuNPs conjugates incubated at the highest temperature yielded the greatest fluorescence increases. Particularly, polyA_20_ DNA−AuNPs and polyA_30_ DNA−AuNPs showed relatively higher thermal stability in that even no dissociation have been observed at lower temperatures. For polyA_20_ DNA, it released only by 0.2% until 65 °C. As for polyA_30_ DNA, desorption happened at 75 °C with a fraction of 1.79%. In addition, we heated these conjugates at 95 °C for 10 minutes, 20 minutes, and 30 minutes (Fig. 1C) to have a further understanding about the thermal stability of these conjugates. Amazingly, all of the conjugates showed a high stability even been exposed at 95 °C for a long time. After heated at 95 °C for 30 min, PolyA_10_ DNA desorbed 39.55%, however polyA_20_ DNA and polyA_30_ DNA only desorbed 25.14% and 16.71% respectively. Serving as an anchoring block, the polyA block was adsorbed onto the AuNPs through every adenine nucleotide to fully take the multivalent advantage. In our experiment, the DNA desorption clearly showed an adenine length-dependent increase and that’s in line with the recent report^47^,^49^.

Moreover, we examined the stability of polyA_20_ DNA−AuNPs containing higher NaCl concentration (0.1 M and 0.3 M). Fractions of released polyA_20_ DNA from AuNPs over a period of 4, 6, 8 and 12 minutes at 95 °C was quantified (Fig. 1D). Although NaCl has been shown to accelerate the desorption for both thiolated DNA and polyA DNA^45^,^50^ and faster desorption was observed with a greater concentration of NaCl^48^. In our result, however, NaCl had little regular effect on the DNA desorption, exhibiting this kind of nanoconjugate was highly resistant to salt and heating.

### Effect of pH on desorption of DNA from the polyA DNA**−**AuNPs conjugates

Since DNA−AuNPs hybrids were always used in numerous of biochemical assays which may take place in different pH buffers, it’s necessary to study the pH effect. Thus, we next study desorption of these conjugates in pH 4, pH 7.4, pH 11, pH 13 in 5 mM HEPES buffer, respectively. To prevent other ions disturbing our results, we used HCl and NaOH to adjust buffer pH cautiously. The buffer pH was adjusted back to pH 7.4 to ensure the accuracy of FAM fluorescence.

**Figure 2:**
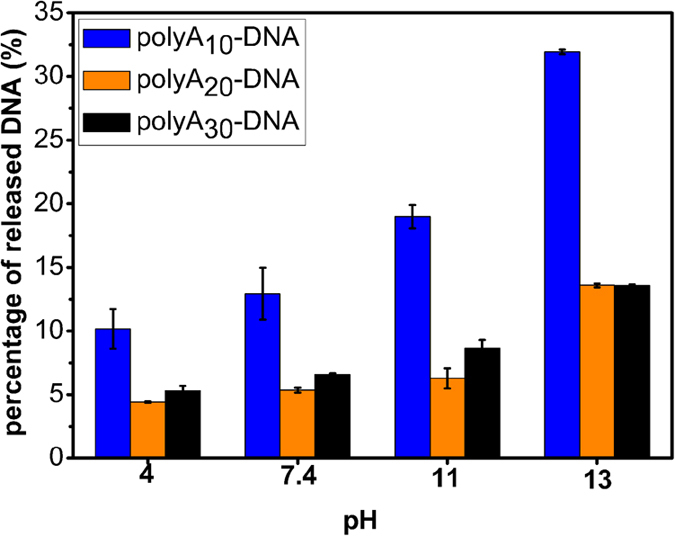
Effect of pH on the release of the polyA (10, 20 and 30, respectively) DNAs into solution. The buffers (5 mM HEPES) were adjusted for pH 4, 7.4, 11 and 13.

As show in Fig. 2, after stored at room temperature for three days, in pH 4, the released DNA remained the lowest, while in pH 11, the amount of release began to rise. Interestingly, polyA_20_ DNA−AuNPs appeared lowest desorption suggesting the highest conjugate stability. It seems like the length of polyA was not a dominant effect on the DNA dissociation any more when the adenine number was beyond 20. Therefore, extreme high pH appeared to favor DNA desorption. Our result is consistent with the previous study about the pH effect on thiolated DNA^45^. Despite the partially dissociation, polyA_20_ DNA−AuNPs and polyA_30_ DNA−AuNPs hybrids still remained a cheerful stablity with a low desorption 13.5% after stored at pH 13 buffer for 3 days.

### Effect of Organic Solvents on desorption of DNA from the polyA DNA**−**AuNPs conjugates

**Figure 3:**
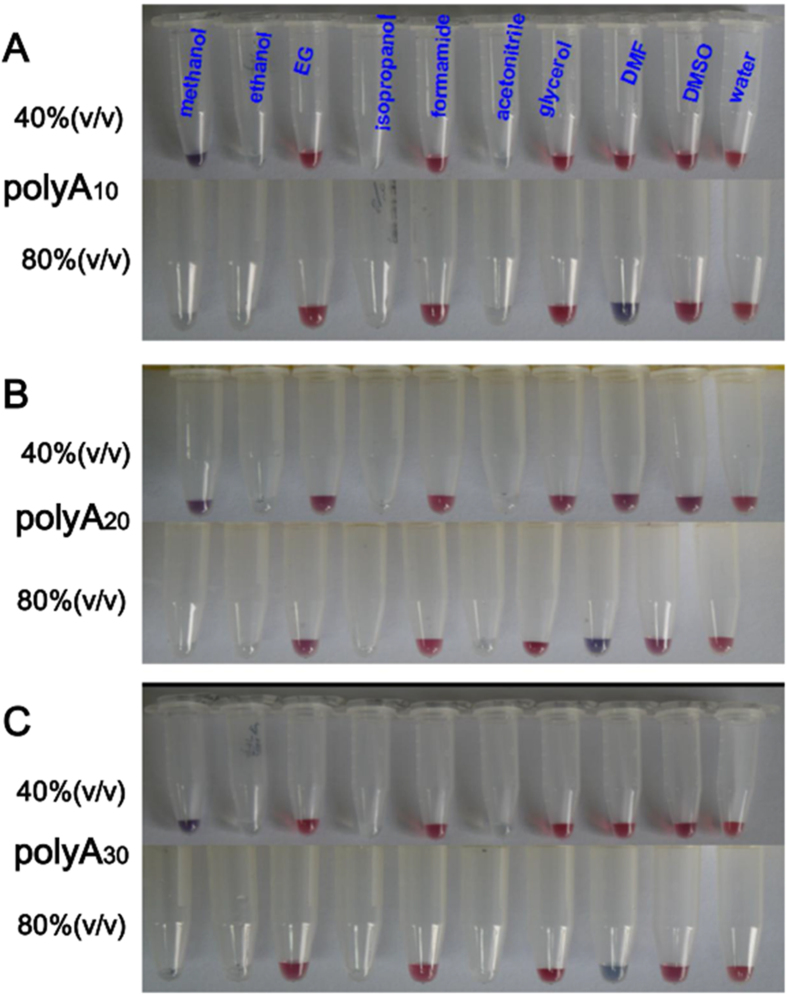
Photographs of polyA (10, 20 and 30, respectively) DNA-functionalized AuNPs dispersed in various concentrations of organic solvents. DMF = dimethylformamide; DMSO = dimethyl sulfoxide; EG = ethylene glycol. Each sample also contained 5 mM HEPES, pH 7.4. (A) Photographs of polyA_10_-DNA-functionalized AuNPs dispersed in various concentrations of organic solvents. (B) Photographs of polyA_20_-DNA-functionalized AuNPs dispersed in various concentrations of organic solvents. (C) Photographs of polyA_30_-DNA-functionalized AuNPs dispersed in various concentrations of organic solvents.

Organic solvents play a vital role during various biochemical operations. Studies on the effect of organic solvents and ionic liquids have been reported previously with the thiolated DNA-AuNPs, and the studies focused on thermodynamic properties and hybridization kinetics of the conjugates mostly^51^,^52^. Here we tested the effect of different organic solvents on the non-thiolated DNA-AuNPs stability. As shown in Fig. 3, the conjugates were stable in 40% (v/v) of the solvents with the exception of ethanol, isopropanol and acetonitrile, which aggregated completely. The aggregation may attribute to the low solubility of DNA in those organic solvents compared to aqueous solution. This result testified that the nanoconjugates can be successfully dispersed in most majority of these organic solvents instead of being damaged. Conjugates in methanol partially aggregated as well, as indicated by the grey colour of dispersed AuNPs. By doubling the solvent concentrations to 80% (v/v), AuNPs aggregation began in DMF, as indicated by the dark blue colour of dispersed AuNPs.

**Figure 4:**
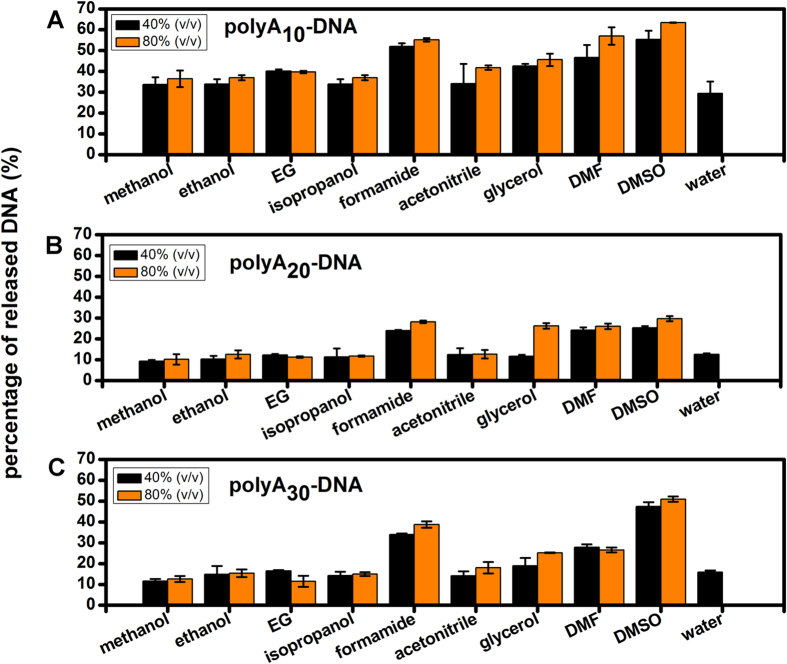
Effect of various concentration (40%, 80%) solvents on the release of the polyA_10_, polyA_20_, polyA_30_-DNA into solution. Each sample also contained 5 mM HEPES, pH 7.6.

However, regardless of the aggregation, we found DNA dissociation still remained at a low standard. Then, we quantified the amount of DNA desorption of DNA−AuNPs dispersed in 40% or 80% of various solvents all contained 5 mM HEPES, pH 7.4. As can be seen in Fig. 4, formamide, DMF, and DMSO harshly intensified the DNA dissociation for all of the 3 kind of hybrids. What’s more, polyA_10_ DNA−AuNPs showed the most unstablity with beyond 30% of the DNA release fraction even in 40% of the solvents after one week of storage at room temperature. Notably, polyA_20_ DNA−AuNPs and polyA_30_ DNA−AuNPs appeared excellent steadiness. The dissociation of DNA was below 20% in most majority of those solvents excepted formamide, DMF, and DMSO. Based on the result as above, most of the organic solvents had a pretty moderate effect on DNA desorption of this kind of nanoconjugates.

### DNA Dissociation Kinetics as a function of polyA length, EDTA, Ethanol and pH

**Figure 5:**
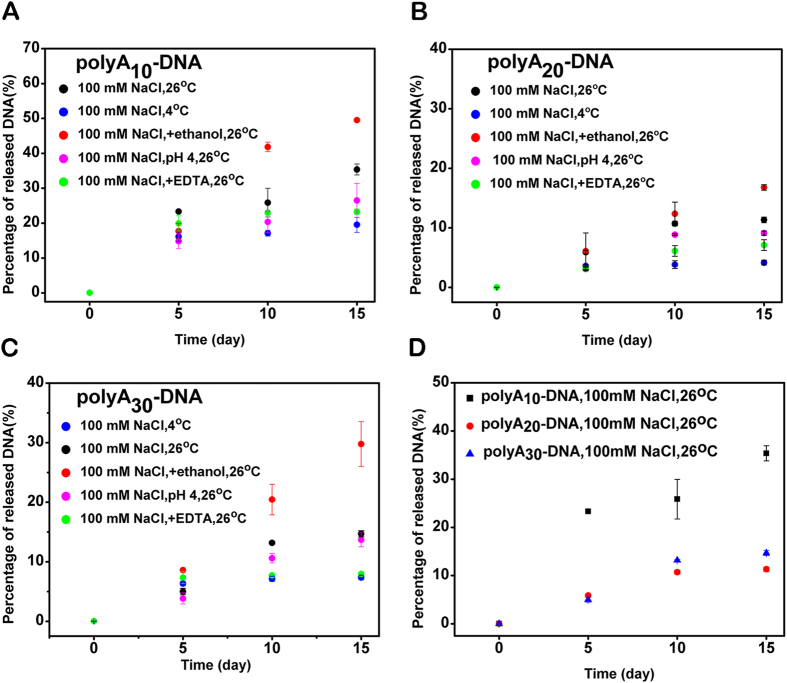
Dissociation of the three polyA_10_, polyA_20_, polyA_30_ DNA from AuNPs surface as a function of DNA sequence, salt, EDTA (2 mM), temperature, and ethanol (80%). All samples contained 100 mM NaCl, pH 7.4. (A, B, C) Dissociation of the polyA_10_ DNA, polyA_20_ DNA and polyA_30_ DNA from AuNPs surface at different storage conditions. (D) Comparison of the DNA desorption kinetics come from the 3 different kinds of DNA AuNPs conjugates. polyA_10_ DNA−AuNPs (black block), polyA_20_ DNA−AuNPs (red dot) and polyA_30_ DNA−AuNPs (blue triangle) contained 100 mM NaCl, in 26 °C.

It has been proved that significant amount of DNA dissociated from the AuNPs surface in a normal storage or reaction buffer for both thiolated DNA and polyA DNA^45^,^48^. However, it was necessary to prepare purified DNA−AuNPs conjugates for different experiments. In this study we also test the DNA desorption as a function of polyA length, EDTA, ethanol and pH systematically. Since 100 mM NaCl is a commonly used salt concentration for DNA−AuNPs storage, this NaCl concentration was selected during our test. As shown in Fig. 5, initial fractions of released DNA were defined close to zero for all samples due to the free DNAs were removed completely after the centrifugation. For the three kind of polyA DNA−AuNPs, ethanol provided no protection on the conjugates, by contraries, ethanol (red dots) accelerated the DNA desorption dramatically (showed in Fig. 5A, B, C). EDTA (green dots), low temperature (blue dots) and low pH (pink dots) seemed have an obvious protection on the conjugates compared to the control (black dots). Particularly, samples with 2 mM EDTA inside yielded less amount of released DNA at 26 °C, suggesting the distinct protection effect of EDTA. EDTA was usually applied to keep DNA from enzymolysis and remove trace amount s of divalent metal ions. This may contribute to protect the conjugates. Moreover, all of the hybrids exhibited cheerful stability stored at room temperature (Fig. 5D). PolyA_10_ DNA (black block) desorbed ~35% after 15 days, while polyA_20_ DNA (red dot) and polyA_30_ DNA (blue triangle) desorbed ~11% and 15% respectively. Thus, if long time storage is needed, a relatively low temperature and slightly acidic buffers with a trifle of EDTA may be useful.

## Conclusion

To sum up, we have systematically studied the dissociation of different adenine length (polyA_10_, polyA_20_, polyA_30_) DNA on gold nanoparticle as function of diverse experimental and storage conditions. By quantifying the dissociation of polyA modified DNAs on gold nanoparticles, we demonstrated that dissociation of DNA was accelerated by high temperature, high salt, alkalin buffer and ethanol. Thermal stability of these polyA DNA−AuNPs varied with the length of the polyA anchors. As polyA anchor lengthened, the DNA−AuNPs showed more resistant to heating. PolyA_20_ DNA−AuNPs and polyA_30_ DNA−AuNPs exhibited preferable endurance after other drastic treatments and preserving conditions. With this study, we have proved that the polyA DNA−AuNPs should be stored in 4 °C and low pH buffer with EDTA. The quantitative data may be helpful to the researchers whose studies employ polyA DNA−AuNPs conjugates, and provide new insights into the essential mechanism of polyA DNA dissociation from the surface of AuNPs.

## Methods

### Materials

AuNPs with 13 nm diameter were synthesized on the basis of the standard citrate reduction procedures^31^. All DNA samples were purchased from Sangon Biotech Co., Ltd (Shanghai, China) and purified by HPLC. The DNA sequences with Carboxyfluorescein (FAM)-labeled are listed in Table 1. HAuCl4 and KCN, EDTA, Sodium chloride, hydrochloric acid, Ethanol, sodium hydroxide, and 4-(2-hydroxyethyl) piperazine-1-ethanesul-fonate (HEPES) were purchased from Sinopharm Chemical Regent Co. Ltd. β-mercaptoethanol (MCH) was purchased from Sigma-Aldrich. All buffer solutions were freshly prepared and filtered, and millipore water was used for all experiments.

### PolyA DNA attachment to AuNPs

PolyA DNA−AuNPs nannoconjugates were prepared using the low pH DNA loading method recently reported by Liu^50^. The general procedure includes four steps. First, a small volume (3 μl) of DNA stock solution (100 μM in 5 mM HEPES buffer, pH 7.4) was added to 200 μL prepared AuNPs solutions (10 nM) and mixed via a brief vortex mixing, and the final DNA concentration was 1.5 μM. Second, 3 μl of 500 mM pH 3 citrate·HCl buffer was added with a brief vortex mixing for a final concentration of 10 mM. Then the sample was incubated at room temperature for 3 minutes. Third, 12 μl of 500 mM HEPES buffer (pH 7.6) was used to adjust the pH of the AuNPs back to neutral. What’s more, another 10 minutes of incubation at room temperature was needed. Finally, the DNA modified AuNPs was washed by repeated centrifuged at 15000 rpm for 20 minutes and the supernatant was removed. The nanoparticles were washed by ~6 rounds of centrifugation and then re-suspended with 5 mM HEPES (pH 7.4) so that the free DNA was removed completely. The purified DNA−AuNPs hybrids were dispersed in 5 mM HEPES buffer for further use.

### Quantitative analysis of released DNA

The purified nanoconjugates were dispersedin appropriate buffers and transferred into different microcentrifuge tubes (20 μl each with AuNPs concentration being ~9 nM). In case of the interferences brought by the oligonucleotides released during storage, conjugates should be purified freshly for experiments. After treatments, the tubes were centrifuged at 12000 rpm for 15 minutes and the supernatant was diluted to 400 μl with HEPES buffer (5 mM, pH 7.4). All sample tubes should be stored at a dark place in that photobleaching may happen on the FAM fluorescein. We quantified the released DNA by using a displacement-based fluorescence method. First, calibration curves were built by plotting the fluorescence intensity of each standard DNA concentration series. Then 20 μl of the purified conjugates (~9 nM) were treated with 100 mM 6-mercapto-1-hexanol (MCH) solution to reach a final concentration of 20 mM. This solution was incubated overnight, and centrifuged at 12000 rpm for 15 minutes. And the supernatant was diluted to 400 μl with HEPES buffer for fluorescence measurement. Since the fluorescence of all these testing supernatant solutions (Ft) and MCH-treated AuNPs (F) were measured, corresponding concentration of testing supernatant solutions (Ct) and MCH-treated AuNPs solution (C) could be calculated according to the calibration curves built before. Thus, the fraction of released DNA was determined to be Ct/C. For estimating the percentage of released DNA, we set 10 μl as a fixed volume of supernatant for fluorescence testing every time. All experiments were run in triplicate. The fluorescence was quantified using a Fluorescence spectrometer (Edin Burgh F900).

### Thermal stability of polyA DNA**−**AuNPs

To study the effect of elevated temperature, each 20 μl of the conjugates (~9 nM, with 5 mM HEPES, pH 7.4) were incubated at designated temperatures with Thermo mixer comfort (Eppendorf).After that, the supernatant fluorescence was measured after a centrifugation at 12000 rpm for 15 minutes.

### Effect of pH on desorption of DNA from the polyA DNA**−**AuNPs conjugates

To study the effect of pH, we adjust the pH of 5 mM HEPES buffer solutions to three different levels (pH 4, 11, and 13) beforehand. The purified conjugates were centrifugated as described above and dispersed in 25 mM HEPES buffer (pH 7.6) with a final AuNPs concentration of 45 nM. 4 μl of such AuNPs was mixed respectively with 100 μl of those buffers above. All samples were stored at room temperature. 3 days later, the supernatant pH value was adjusted to 7.4 with a final volume of 400 μl before its fluorescence was measured since FAM is a pH-dependent fluorophore. A centrifugation was needed to remove the free DNA before the measurement.

### Effect of organic solvents on desorption of DNA from the polyA DNA**−**AuNPs conjugates

The purified conjugates were centrifugated as described above and dispersed in 25 mM HEPES buffer (pH 7.6) with a final AuNPs concentration of 45 nM. 4 μl of such AuNPs was mixed respectively with 16 μl 100% and 50% solvents so that the final solvent concentration was 80% and 50%, respectively, and the AuNPs concentration was ~9 nM. All these samples were exposed at room temperature for one week. To test the effect of the organic solvents, the colour of the AuNPs was recorded by a digital camera. Then, the supernatant fluorescence was measured after a centrifugation at 12000 rpm for 15 minutes.

### DNA dissociation at different storage conditions

To study the DNA dissociation at different storage conditions, purified DNA−AuNPs hybrids were stored at various environmental and buffer conditions. Each 20 μl of the conjugates (~9 nM) in buffer containing 100 mM NaCl, 5 mM HEPES, pH 7.4, After stored at designated time points (0, 5, 10, 15 days) , the supernatant fluorescence was measured with a centrifugation at 15000 rpm for 10 minutes.

## Author Contributions

D.P. and Y.Z. designed the experiments. W.L., L.W., J.L. and Z.Z. performed experiments and data analysis. W.L., J.L., J.S., X.Z. and D.P. performed the calculations. D.P. and Y.Z. led the whole work and the analysis. W.L. and D.P. wrote the text. All authors reviewed the manuscript.

## Additional Information

**How to cite this article**: Lu, W. *et al.* Quantitative investigation of the poly-adenine DNA dissociation from the surface of gold nanoparticles. *Sci. Rep.* **5**, 10158; doi: 10.1038/srep10158 (2015).

## Figures and Tables

**Table 1:** A List of the DNA Sequences Used in This Work.

**DNA Name**	**Sequences (from 5’to3’)**	**Modification**
polyA_10_-DNA	AAAAAAAAAAACCATTCCCACCCTT	5’FAM
PolyA_20_-DNA	AAAAAAAAAAAAAAAAAAAAACCATTCCCACCCTT	5’FAM
polyA_30_-DNA	AAAAAAAAAAAAAAAAAAAAAAAAAAAAAAACCATTCCCACCCTT	5’FAM
